# Diagnostic Performance of Combined Conventional CT Imaging Features and Radiomics Signature in Differentiating Grade 1 Tumors from Higher-Grade Pancreatic Neuroendocrine Neoplasms

**DOI:** 10.3390/cancers17061047

**Published:** 2025-03-20

**Authors:** Florent Tixier, Felipe Lopez-Ramirez, Alejandra Blanco, Ammar A. Javed, Linda C. Chu, Ralph H. Hruban, Mohammad Yasrab, Daniel Fadaei Fouladi, Shahab Shayesteh, Saeed Ghandili, Elliot K. Fishman, Satomi Kawamoto

**Affiliations:** 1 The Russell H. Morgan Department of Radiology and Radiological Science, School of Medicine, Johns Hopkins University, Baltimore, MD 21205, USA; tixier@jhu.edu (F.T.); jlopezr5@jh.edu (F.L.-R.); ablanco5@jhmi.edu (A.B.); lchu1@jhmi.edu (L.C.C.); myasrab1@jh.edu (M.Y.); fouladi.daniel@yahoo.com (D.F.F.); shahabshayesteh@gmail.com (S.S.); saeed.ghandili@gmail.com (S.G.); efishman@jhmi.edu (E.K.F.); 2 Department of Surgery, The NYU Grossman School of Medicine and NYU Langone Health, New York, NY 10016, USA; ammar.javed@nyulangone.org; 3 The Sol Goldman Pancreatic Cancer Research Center, Department of Pathology, School of Medicine, Hopkins University, Baltimore, MD 21205, USA; rhruban@jhmi.edu; 4 Sidney Kimmel Comprehensive Cancer Center, School of Medicine, Johns Hopkins University, Baltimore, MD 21205, USA

**Keywords:** pancreatic neuroendocrine tumor, pancreatic neuroendocrine neoplasm, pathologic grade, radiomics, machine learning algorithms, support vector machine model, tumor grade prediction

## Abstract

Background/Objectives: Accurate identification of grade 1 (G1) pancreatic neuroendocrine tumors (PanNETs) is crucial due to their rising incidence and emerging nonsurgical management strategies. This study evaluated whether combining conventional CT imaging features, CT radiomics features, and clinical data improves differentiation of G1 PanNETs from higher-grade tumors (G2/G3 PanNETs and pancreatic neuroendocrine carcinomas [PanNECs]) compared to using these features individually. Methods: A retrospective analysis included 133 patients with pathologically confirmed PanNETs or PanNECs (70 males, 63 females; mean age, 58.5 years) who underwent pancreas protocol CT. A total of 28 conventional imaging features, 4892 radiomics features, and clinical data (age, gender, and tumor location) were analyzed using a support vector machine (SVM) model. Data were divided into 70% training and 30% testing sets. Results: The SVM model using the top 10 conventional imaging features (e.g., suspicious lymph nodes and hypoattenuating tumors) achieved 75% sensitivity, 81% specificity, and 79% accuracy for identifying higher-grade tumors (G2/G3 PanNETs and PanNECs). The top 10 radiomics features yielded 94% sensitivity, 46% specificity, and 69% accuracy. Combining all features (imaging, radiomics, and clinical data) improved performance, with 94% sensitivity, 69% specificity, 79% accuracy, and an F1-score of 0.77. The radiomics score demonstrated an AUC of 0.85 in the training and 0.83 in the testing set. Conclusions: Conventional imaging features provided higher specificity, while radiomics offered greater sensitivity for identifying higher-grade tumors. Integrating all three features improved diagnostic accuracy, highlighting their complementary roles. This combined model may serve as a valuable tool for distinguishing higher-grade tumors from G1 PanNETs and potentially guiding patient management.

## Simple Summary

The increasing detection of low-grade pancreatic neuroendocrine tumors has led to growing interest in non-surgical management, making accurate identification of grade 1 (G1) tumors essential. This study investigates whether combining conventional CT imaging, radiomics, and clinical data improves differentiation between low-grade (G1) and higher-grade tumors compared to using these features individually. A retrospective analysis of 133 patients with confirmed pancreatic neuroendocrine tumors or carcinomas was conducted using a support vector machine model to assess imaging, radiomics, and clinical features. Conventional CT imaging features showed higher specificity, while radiomics exhibited greater sensitivity for identifying higher-grade tumors. Combining all features improved diagnostic accuracy, highlighting their complementary roles. These findings suggest that integrating all three features can enhance tumor classification, potentially guiding treatment decisions and patient care. This approach offers a more effective, noninvasive method for tumor grading, which may support the development of personalized management strategies.

## 1. Introduction

Pancreatic neuroendocrine neoplasms (PanNENs) are rare pancreatic tumors. Their incidence and prevalence have been steadily increased, partially due to increased diagnosis of early-stage disease owing to the widespread use of cross-sectional imaging [1,2]. Currently, PanNENs account for 2–5% of all pancreatic neoplasms [1,3]. The high heterogeneity of PanNENs makes standardization of therapeutic strategies and optimal management difficult [4], and the management of PanNENs largely depends on disease stage and grade [5]. Under current guidelines, patients with T1-stage pancreatic neuroendocrine tumors (PanNETs) may undergo active surveillance or surgical resection [6,7,8]. While multiple treatment options exist, surgical resection is typically indicated for localized tumors that are larger than 1–2 cm, grade 2 or higher, symptomatic, functional, or associated with regional lymph node metastasis in patients eligible for pancreatectomy [8,9].

PanNENs exhibit varying degrees of biological aggressiveness, which is reflected in their histological grade [10]. They are classified using the WHO grading system, which is based on the assessment of proliferative activity in the tumor, and serves as an independent prognostic factor for survival and treatment planning [11]. Well-differentiated PanNENs, termed pancreatic neuroendocrine tumors (PanNETs), are categorized based on their Ki-67 labeling index or mitotic count: Grade 1 (G1): Ki-67 < 3% and <2 mitoses/10 high-power field (HPF); Grade 2 (G2): Ki-67 3–20% or 2–20 mitoses/10 HPF; and Grade 3 (G3): Ki-67 > 20% or >20 mitoses/10 HPF. Pancreatic neuroendocrine carcinomas (PanNECs) are defined by their high-grade morphology, and they can have either a small cell carcinoma or large cell carcinoma morphology. Identifying G1 PanNETs is crucial because small tumors can sometimes be safely observed.

Contrast-enhanced CT features of PanNENs have been reported to help distinguish low-grade (G1) PanNETs from higher-grade PanNENs, including G2/G3 PanNETs and PanNECs. Reported CT features of higher-grade PanNENs include tumor size greater than 2.0 cm, ill-defined tumor margins, vascular involvement, calcification, pancreatic or biliary ductal dilatation, and lymph node or liver metastasis [10,11,12,13]. Hypoenhancement of PanNENs on contrast-enhanced CT has also been linked to higher-grade PanNENs [11,13,14].

Radiomics is a relatively new noninvasive approach for image analysis, allowing the extraction of great amounts of quantitative imaging features from clinical images and used as input data in artificial intelligence (AI) technique [15]. Radiomics is a rapidly evolving field and has primarily been exploited in oncological applications, including better tumor characterization, staging, prediction of patient outcomes, and treatment response [16,17]. In pancreatic imaging, radiomics has been applied to tumor characterization, survival prediction, and tumor response evaluation. For PanNENs, radiomics has been applied to predict tumor grading with promising results [12,18,19,20,21].

We hypothesize that combining conventional CT imaging features, CT radiomics features, and clinical data (patient age, gender, and tumor location) can enhance the differentiation of higher-grade PanNENs (including G2 and G3 PanNETs and PanNECs) from G1 PanNETs compared to using either conventional CT imaging or radiomics features alone or combined with clinical data. The purpose of the study is to determine whether integrating conventional CT imaging features, radiomics features, and clinical data can improve the differentiation of higher-grade PanNENs from G1 PanNETs using a support vector machine (SVM) model.

## 2. Materials and Methods

### 2.1. Patients

The study was approved by the Institutional Review Board. The need for obtaining consent was waived. A total of 133 patients with pathologically confirmed PanNEN (70 males, 63 females) with a mean age of 58.5 ± 13.1 years (range: 21–83) who had dedicated pancreas protocol CT between 2012 and 2019 were retrospectively identified and evaluated in this study.

### 2.2. CT Acquisition

All CT images were acquired using a dual-source MDCT scanner (Somatom Definition Flash or Somatom Drive, Siemens Medical Solutions USA, Inc., Malvern, PA, USA). Typical scan parameters included a tube voltage of 120 kVp, a quality reference of 290 mAs for the online dose modulation system (CareDose4D; Siemens Medical Solutions, USA, Inc., Malvern, PA, USA), a pitch of 0.6, and a collimation of 128 × 0.6 mm. However, the scan protocol was customized for each patient to minimize radiation dose.

The patients were injected with 120 mL of nonionic contrast material (Iohexol [Omnipaque 350, GE Healthcare, Princeton, NJ, USA] or Iodixanol [Visipaque 320, GE Healthcare, Princeton, NJ, USA]) intravenously through a peripheral venous line at an injection rate of 4–5 mL/s. Dual-phase CT protocol included arterial phase timed by fixed delay typically 30 s post-injection or bolus tracking (Siemens Medical Solutions, USA, Inc., Malvern, PA, USA) at 230 HU in the abdominal aorta, and venous phase imaging was performed approximately 60–70 s post-injection. All images were reconstructed into two sets of thin (0.75 mm slice thickness, 0.5 mm increment) and thick (3 mm slice thickness, increment) reconstructions, and these datasets were used for feature extraction.

### 2.3. Image Segmentation

**Figure 1:**
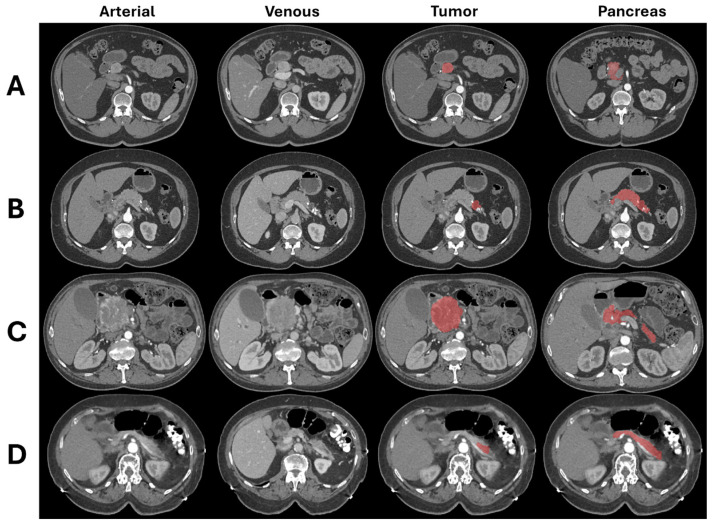
Example cases that are correctly classified by the combined model. (**A**) G1 PanNET (3.5 cm in head). Arterial and venous phase images, segmentation of tumor (red: third column), and entire pancreas (red: fourth column). Note markedly atrophic pancreatic parenchyma. This case was misclassified by the radiomics features model and correctly classified by the conventional imaging features and combined models. (**B**) G1 PanNET (1.5 cm in tail). This case was misclassified by the conventional imaging features model and correctly classified by the radiomics features and combined models. (**C**) G2 PanNET (8.1 cm in head). This case was misclassified by the radiomics features model and correctly classified by the conventional imaging features and combined models. (**D**) G2 PanNET (1.7 cm in tail). This case was misclassified by the conventional imaging features model and correctly classified by radiomics features and combined models.

Manual segmentation of PanNETs and the entire volume of the pancreas was performed as 3D volumes in both arterial and venous phases using thin-slice reconstruction images (Figure 1). Four trained researchers (with 1–4 years of experience) conducted the manual segmentation in both arterial and venous phases using commercially available software Velocity AI^TM^ (Varian Medical Systems, Palo Alto, CA, USA) version 4.1. Three abdominal radiologists, each with 5–30 years of experience after body imaging fellowship training, reviewed and verified the segmentation boundaries on each CT examination. Any necessary corrections were made through face-to-face sessions between the researchers and radiologists.

### 2.4. Image Analysis

#### 2.4.1. Conventional CT Imaging Features

From CT images, 28 conventional CT imaging features, comprising 13 conventional image characteristics and 15 enhancement patterns of the tumors, were included (Table 1). The conventional CT imaging characteristics were evaluated by two abdominal radiologists independently without knowledge of tumor grade. When there was disagreement between two radiologists, a consensus was obtained, and the final decision was determined. Subjectively, tumors were classified as hypoattenuating or iso-/hyperattenuating to normal pancreatic parenchyma in arterial and venous phases, and tumor margin was subjectively categorized as either well-defined or ill-defined. The presence of main pancreatic duct (MPD) dilatation (>3 mm) upstream to the tumor, common bile duct (CBD) dilatation (>1 cm by pancreatic head tumor or CBD stent), subjective upstream pancreatic atrophy, exophytic tumor (>50% of tumor volume protruding from the expected pancreatic contour), liver or abdominal lymph node metastasis (lymph nodes with enlargement, abnormal morphology, or enhancement) on CT, involvement of major peripancreatic vessels (compression, narrowing, encasement, or tumor thrombus), and calcification within the tumor were recorded if present. If the tumor is cystic (containing well-defined area(s) of homogeneous fluid attenuation) or necrotic (containing ill-defined nonenhancing area(s) of low attenuation), these features were recorded.

Enhancement patterns of tumors were evaluated by measuring CT attenuation (Hounsfield units [HU]) of the solid component of the tumor and normal pancreatic parenchyma using region of interests (ROIs) in arterial and venous phases excluding large vessels, calcifications, apparent cystic or necrotic areas of tumor measured by one radiologist. Similarly, CT attenuation of abdominal aorta (arterial and venous phases) and main portal vein (venous phase) were measured by placing ROI in three consecutive slices, and an average of three measurements was used for analysis. In addition to CT attenuation of tumor and normal pancreatic parenchyma, ratio and difference of CT attenuation of tumor relative to the normal pancreas, aorta, and portal vein in arterial and venous phases obtained in following formula were used: Tumor^(Arterial)^/Aorta^(Arterial)^, Tumor^(Venous)^/Aorta^(Venous)^, Tumor^(Arterial)^/Pancreas^(Arterial)^, Tumor^(Venous)^/Pancreas^(Venous)^, Tumor^(Venous)^/Portal vein^(Venous)^, Tumor^(Arterial)^/Portal vein^(Venous)^, Tumor^(Arterial)^/Tumor^(Venous)^, and Tumor^(Arterial)^-Tumor^(Venous)^. These features are listed in Table 1 and also used as conventional CT imaging features for analysis.

#### 2.4.2. Radiomics Features

To achieve an isotropic voxel size of 1 mm^3^, the images and segmented volumes were resampled before conducting radiomics feature extraction. For this preprocessing, the c3d tool in ITK-SNAP [22] was used to employ trilinear interpolation for the images and nearest neighbor interpolation for the segmentation masks.

A total of 4892 radiomics features were then extracted from CT on arterial (2446 features) and venous phase (2446 features) for both PanNET volumes (2446 features) and pancreas volumes (2446 features) using the Pyradiomics package (v3.0.1) [23]. For each image (arterial/venous) and segmentation volume (PanNET/pancreas), a total of 14 shape descriptors and 93 histogram and texture features were extracted. These included 18 histogram features, 24 gray-level co-occurrence matrix (GLCM) features, 14 gray-level differences matrix (GLDM) features, 16 gray-level run-length matrix (GLRLM) features, 16 gray-level size-zone matrix (GLSZM) features, and 5 neighbor gray-tone difference matrix (NGTDM) features). Additionally, histogram and texture features from images transformed by 12 different convolutional filters were also extracted, resulting in a total of 1116 features (93*12 = 1116 features). The employed convolutional filtering techniques included a Wavelets transform (Coiflets 3 wavelets transform with low-pass (L) and high-pass (H) filtering in three spatial coordinates, resulting in eight sub-bands: LLL, LLH, LHL, LHH, HLL, HLH, HHL, and HHH) and Laplacian of Gaussian (LoG, with sigma values of 1, 3, 5, and 10 mm). The radiomic features extraction followed the guidelines outlined by the Image Biomarker Standardization Initiative (IBSI) [24,25], incorporating a bin width of 25 HU, a 26-connectivity for GLSZM and GLCM, and a distance of 1 mm for GLCM, GLDM, and NGTDM. In the following, we refer to radiomics features extracted from images post-convolutional filtering as edge features, given that image convolution enhances edges within the images.

#### 2.4.3. Clinical Features

Patient’s age and gender were assessed as clinical features. Tumor location recorded in the pathology report was also included as a clinical feature in this analysis.

### 2.5. Statistical Analysis and Modeling

We used Python (v3.11.3) with the scipy and scikit-learn (sklearn) libraries to perform the statistical analyses and implement predictive models.

Prior to modeling, we used mutual information to select 100 radiomic features from those derived from convolutional filtered images, using SelectKbest algorithm. This step was done to prevent having a large number of features from the convolutional filtered images (*n* = 4464) in comparison to features from the original images (*n* = 428). The total number of radiomics features considered for the modeling was 528. Patient data were split into 70% for training and 30% for testing. This split was achieved through a stratified sampling method, ensuring an equivalent proportion of tumor grade in both sets. The training set was upsampled using RandomOverSampler from the imbalanced-learn (v0.11) Python library to address the imbalance between the proportion of G1 cases versus higher-grade tumors by adding repeated instances of high-grade tumors to balance the dataset (Table 2). The enhancement patterns that are non-categorical conventional CT imaging features (Table 1) and radiomics features were normalized to a [0–1] scale using minimum and maximum values in the training set. Consequently, normalized features in the testing set may fall outside the [0–1] range.

For radiomics, a LASSO feature selection was applied to the training set for binomial classification of tumor grades, tuning the regularization parameter alpha to select 10 features. The process was conducted using a 10-fold cross-validation, and a cutoff of 10 features was chosen to avoid overfitting, in line with the common practice of using roughly the square root of the number of patients as an upper limit. For conventional CT imaging features, mixing categorical (image characteristics assessed by radiologists) and continuous features (enhancement patterns), we used a recursive feature elimination with a logistic regression model to select the 10 most important features.

Then, we built SVM models for tumor grade classification using (1) conventional CT imaging features, (2) radiomics features, (3) conventional CT imaging features + clinical data, (4) radiomics features + clinical data, and (5) conventional CT imaging features + radiomics features + clinical data as input. A SVM classifier was used with a linear kernel; regularization parameter (C) was set to 1.0 and with a 5-fold cross-validation to assess the robustness of the SVM models. For comparison, we also used a classification model based on a radiomics score, defined as a linear combination of selected radiomics features weighted by their contributions from the LASSO. Models were assessed using performance metrics such as accuracy, specificity, sensitivity, precision, and F1 score. These metrics were computed for the training and testing sets, as well as for small (≤2 cm) tumors on the testing set because tumor size is a well-known predictor of PanNET grade [11,12,13]. The 95% confidence intervals (95% CI) were determined through a bootstrapping approach with 1000 iterations. Differences between continuous variables were analyzed using the Mann–Whitney U test, while categorical variables were compared using the chi-square test. All statistical tests conducted in this study considered *p*-values < 0.05 as indicative of significance.

## 3. Results

### 3.1. Demographics of PanNEN Patients

Among the 133 patients, 4 patients had more than 2 PanNETs (1 patient had 3 PanNETs [1 G1 and 2 G2 PanNETs], and the other 3 patients had 2 G1 PanNETs). Therefore, 138 PanNENs were evaluated. There were 84 patients with G1 PanNET only (44 males, 40 females). A total of 44 patients had G2 PanNETs, including 1 patient who had both G1 and G2 PanNETs (22 males, 22 females) and 1 patient with G3 PanNET (1 male). In total, 4 patients had PanNEC (3 males, 1 female).

### 3.2. Surgery and Pathology Results

All except 2 patients had surgical resection of PanNETs. Two patients with G1 (*n* = 1) and G2 PanNET (*n* = 1) were diagnosed by endoscopic ultrasound and fine needle aspiration. The biopsied patient with a G1 PanNET (1.5 cm) was followed up by CT, and the PanNET was stable for at least 4 years. The remaining patients had Whipple surgery (*n* = 40), distal pancreatectomy with or without splenectomy (*n* = 78), excision/enucleation/central pancreatectomy (*n* = 10), or total pancreatectomy (*n* = 3). The interval between CT and pathological diagnosis was 0.64 ± 3.7 months (range: 0.2–41.7).

The mean pathological tumor size of all PanNEN was 3.1 ± 2.4 cm (range: 0.5–12.5 cm; median: 2.2 cm). The mean size of the G1 PanNETs was 2.5 ± 2.3 cm (median: 1.8 cm), and the mean size of the G2, G3 PanNETs and PanNEC was 4.1 ± 2.3 cm (median: 3.9 cm). Location of the tumor was head (29%), uncinate (3%), neck (1%), body (18%), tail (48%), or diffuse (1%). Among 45 G2 PanNETs in 44 patients, Ki-67 index was available for 43 tumors. (In 2 tumors, it was not available in pathology record.) In all, 9 tumors were Ki-67 index <5%, 20 tumors were ≥5 to <10%, 7 tumors were ≥10% and <15%, and 7 tumors were ≥15% and ≤20%.

### 3.3. SVM Model Performance Using CT Features and Enhancement Patterns of PanNEN

**Figure 2:**
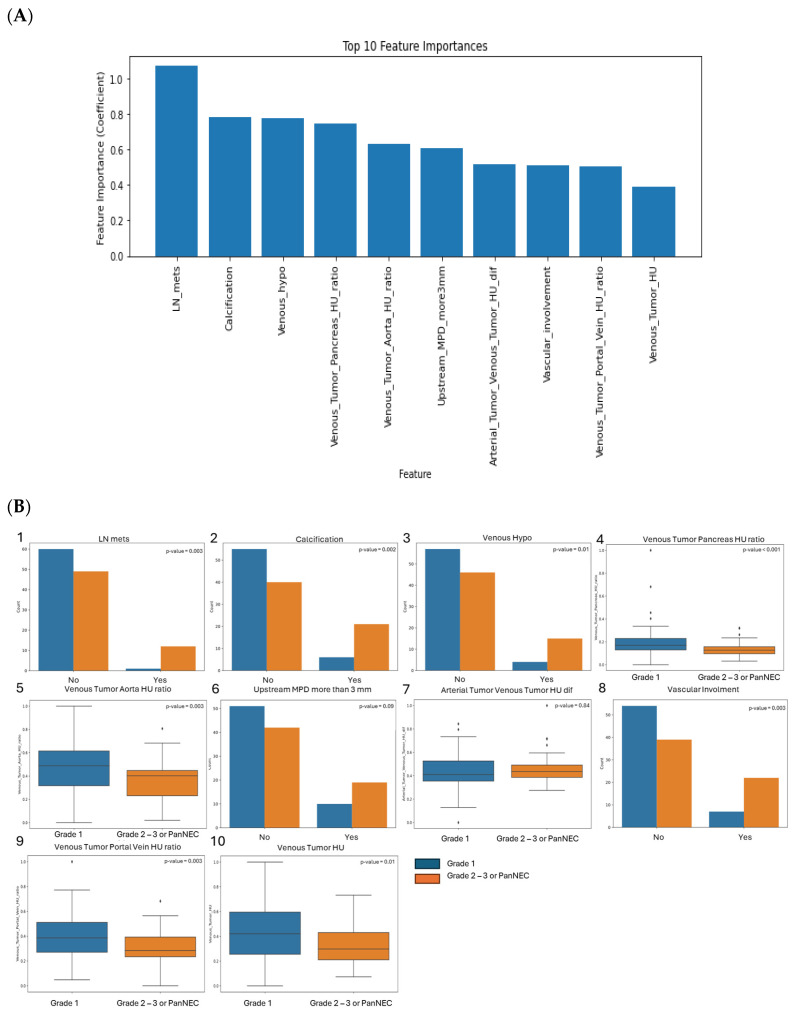
(**A**) Top 10 important conventional CT imaging features to differentiate grade 1 versus higher-grade PanNENs. (**B**) Boxplots and histograms of the 10 selected conventional CT imaging features showing the class repartition or the value of the features for grade 1 versus higher-grade tumors. The reported *p*-values are obtained using the Mann–Whitney U test for continuous variables and the chi-square test for categorical variables.

Among conventional CT imaging features, the top 10 most important features to differentiate G1 versus higher-grade PanNEN are shown in Figure 2A. The feature with the highest importance was lymph node metastasis, followed by presence of calcification, subjectively hypoattenuating tumor relative to normal pancreatic parenchyma in venous phase assessed by radiologists, HU ratio of tumor to pancreas parenchyma in venous phase, HU ratio of tumor to abdominal aorta in venous phase, upstream MPD dilatation greater than 3 mm, difference of HU values of tumor between arterial and venous phases, vascular involvement, HU ratio of tumor to portal vein in venous phase, and HU value of tumor in venous phase. Enhancement patterns were found significantly lower in high-grade neoplasms with the exception of difference of HU values of tumor between arterial and venous phases where no significative difference was found (Figure 2B). For categorical variables, we found higher-grade neoplasms were more subject to have lymph node metastasis, calcification, an hypoattenuated tumor relative to normal pancreatic parenchyma in venous phase, and an upstream MPD dilatation greater than 3 mm.

**Figure 3:**
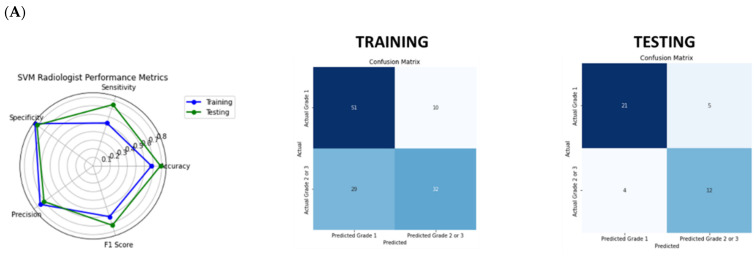

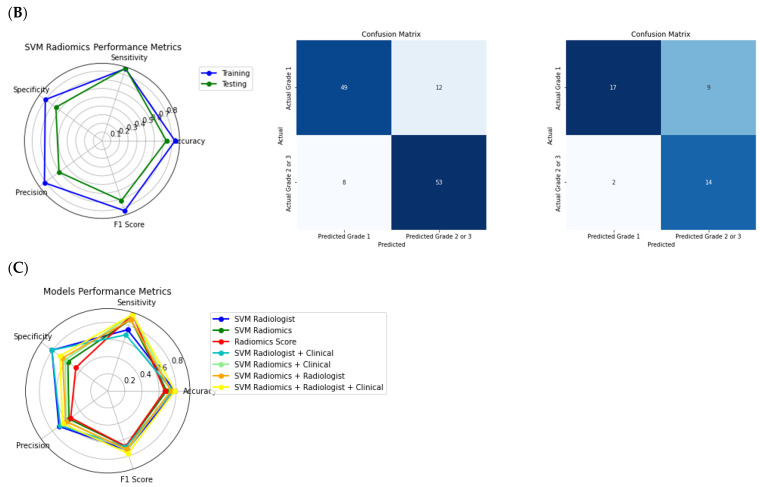
(**A**) SVM model performance using conventional CT imaging features. (**B**) SVM model performance using CT radiomics features. (**C**) Overall SVM performance values for different models (conventional CT imaging features only, CT radiomics features only, combined conventional CT imaging features, CT radiomics features, and clinical data). SVM tadiologist: Conventional CT imaging features assessed by radiologists. Clinical: Clinical data (gender, age, and tumor location).

These features were used to build a prediction model using a SVM model for a testing dataset. There was high specificity (81%; 95% CI: 0.64–0.95) and reasonable sensitivity (75%; 95% CI: 0.53–0.94) to predict higher-grade PanNEN (greater than grade 1), with overall accuracy of 79% (95% CI: 0.67–0.90) (Table 3, Figure 3A). When clinical features (age, gender, and tumor location) were added to conventional CT imaging features into the model, there was no improvement of the performance (Table 3, Supplementary Figure S1A).

### 3.4. ROC Curve Analysis and SVM Model Performance Using Radiomics Features

Using mutual information and the SelectKBest algorithm, we reduced the number of edge features to 100, and details of the selected edge features are displayed in Supplementary Figure S2.

**Figure 4:**
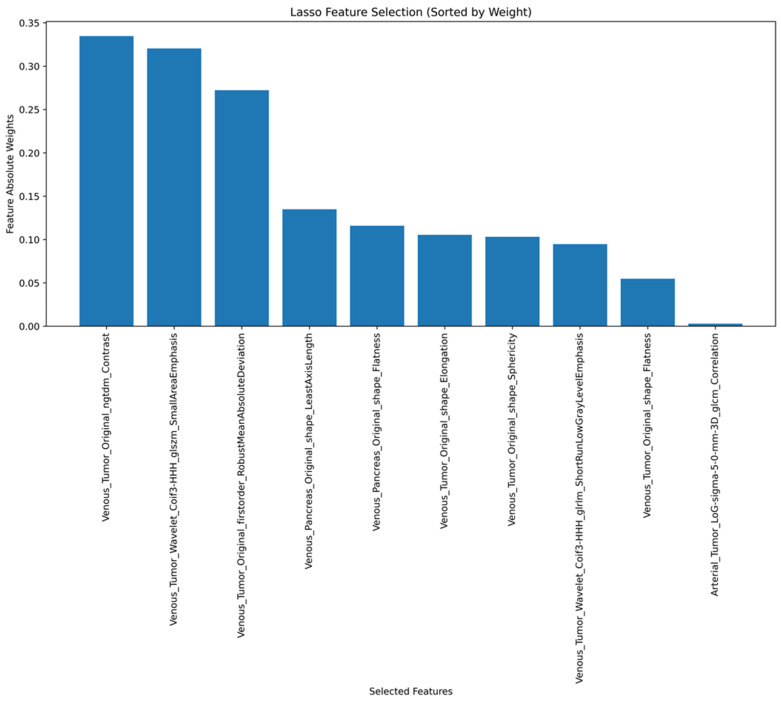
Top 10 radiomics features in the model selected by the LASSO feature selection based on a total of 528 features, including 100 edge features and 428 radiomics features from the original images.

A LASSO feature selection was conducted based on a total of 528 features, including 100 edge features and 428 radiomics features from the original images to choose 10 radiomics features. Out of these top 10 radiomics features, 8 features are derived from the tumor, while 2 are from the full pancreas; 3 are edge features, and 7 originate from the original images. Only one selected feature is associated with the arterial phase (Figure 4).

**Figure 5:**
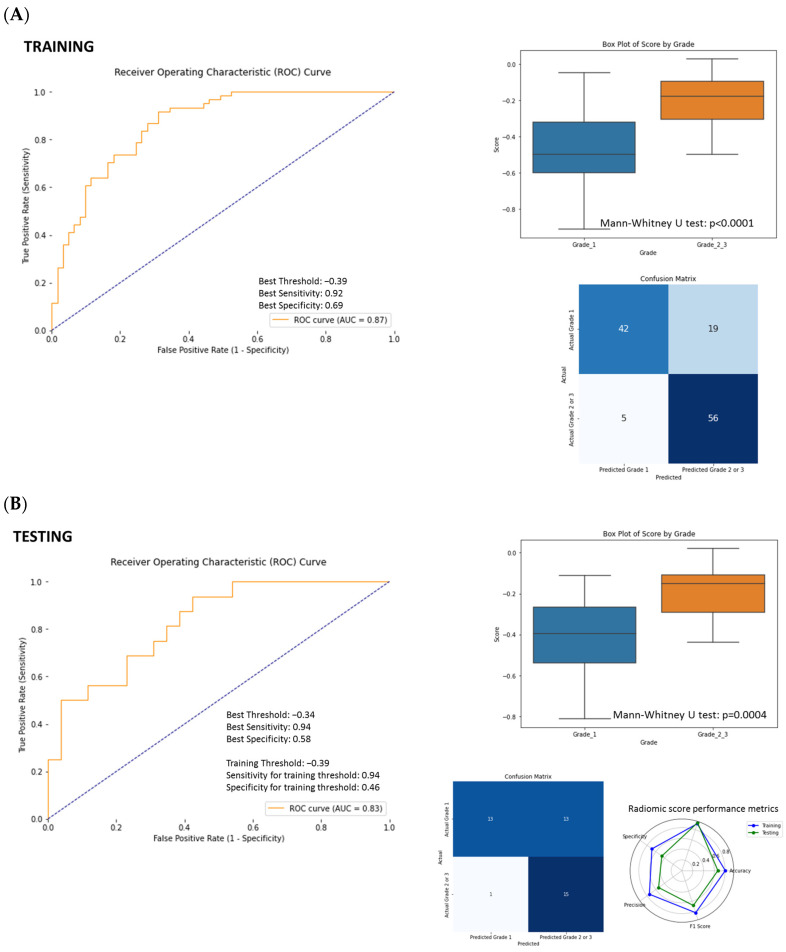
(**A**) Receiver operating characteristic (ROC) curve analysis on the training set to determine the optimal cutoff value (Youden J Index) to assess the classification performance of the radiomics score model. (**B**) ROC curve analysis on the testing set. AUC of ROC curve in testing set was 0.83.

A linear combination of the selected features and their respective weights was used to generate a radiomics score. Then, the radiomics score was utilized to plot the receiver operating characteristic (ROC) curve in both the training and testing sets and selected the Youden J Index to assess the classification performance of the model (Figure 5). The area under the curve (AUC) of the ROC in the training set was 0.85 and in the testing set was 0.83 (Figure 5). The sensitivity, specificity, and accuracy of the radiomics score to predict higher-grade PanNENs (greater than grade 1) were 94% (95% CI: 0.80–1.0), 46% (95% CI: 0.34–0.74), and 69% (95% CI: 0.54–0.83) in the testing set (Table 3).

The same set of 10 selected features was applied to a SVM model, and a higher sensitivity, lower specificity, and accuracy to predict higher-grade PanNENs (sensitivity 88% (95% CI: 0.69–1.0), specificity 58% (95% CI: 0.38–0.77), and accuracy 69% (95% CI: 0.55–0.83)) compared to conventional CT imaging features were obtained (Figure 3B). Adding clinical data to radiomics features in the SVM model led to a slight enhancement in the classification performance with a sensitivity of 94% (95% CI: 0.79–1.0), a specificity of 62% (95% CI: 0.42–0.79), and accuracy 74% (95% CI: 0.62–0.86) (Supplementary Figure S1B, Table 3).

The SVM model combining conventional CT imaging features, radiomics features, clinical data, sensitivity, specificity, and accuracy to predict higher-grade PanNENs were 94% (95% CI: 0.78–1.0), 69% (95% CI: 0.52–0.87), and 79% (95% CI: 0.64–0.90), respectively, with the highest F1-score (0.77; 95% CI: 0.60–0.91) (Table 3). Overall, combining radiomics, conventional CT imaging features, and clinical data together appears to be the best option, yielding almost perfect sensitivity and reasonable specificity. Similar results were obtained for small (≤2 cm) tumors (Supplementary Figure S3); however, it was based on only 16 small tumors with only 3 higher-grade tumors in testing set. Overall performance values for the different modes are shown in Figure 3C and summarized in Table 3.

## 4. Discussion

PanNENs are classified based on their degree of differentiation (well-differentiated PanNETs vs. PanNECs), and well-differentiated PanNETs are graded based on the mitotic rate and Ki67 labeling index as G1, G2, or G3 [3]. Histological tumor grade is an important factor to dictate aggressiveness of the PanNENs, and when reviewing studies, it is important to note which grading scheme was employed. The 2010 WHO classification used mitotic rate and Ki67 labeling and classified PanNENs as low-grade (G1), intermediate-grade (G2), and high-grade (G3). However, in the 2019 update of the WHO classification, G3 tumors were separated into two groups. Well-differentiated tumors with high mitotic rates and Ki67 index were designated G3 PanNETs, while poorly differentiated neoplasms were designated PanNECs. In the 2019 WHO classification, PanNECs are high-grade carcinomas that resemble small cell carcinoma or large cell neuroendocrine carcinoma of the lung [3,26]. In addition to TNM stage, tumor grades of PanNETs are the important prognostic factors. Reported 5-year survival rates for G1, G2, and G3 tumors were 75%, 62%, and 7%, respectively, and stage I, II, III, and IV tumors using AJCC/UICC classification were 92%, 84%, 81%, and 57%, respectively [27].

The treatment strategies for patients with a PanNET have been evolving, with increasing incidence of incidentally detected small PanNETs. Surgical indications of small (<2 cm) asymptomatic sporadic nonfunctioning PanNETs have recently been reconsidered, and nonsurgical management can be considered [4]. In addition to the size of the tumor, the determination of selective observation versus surgical resection is based on individualized decisions based on tumor grade, age, comorbidities, tumor location, radiological characteristics, surgeon, and patient preference [28]. Therefore, accurate differentiation of G1 PanNETs from higher-grade PanNENs is of great clinical significance.

Prior studies reported several conventional CT imaging features that can help to predict tumor grade of PanNENs. Galliot et al. found that the size, presence of pancreatic duct dilatation, and vascular invasion were correlated with the presence of high-grade in incidentally found PanNETs [29]. Canellas et al. reported that tumor size greater than 2.0 cm, presence of vascular involvement, presence of pancreatic duct dilatation, and presence of lymphadenopathy are the CT features predictive of G2/3 tumors classified by the 2010 WHO classification [12]. Kim et al. reported that <1.1 enhancement ratio of PanNET relative to pancreatic parenchyma, poorly defined margin, tumor size > 3 cm, bile duct dilatation, and vascular invasion were associated with G3 tumors distinguished from G1/2 tumors based on the 2010 WHO classification [13]. Takumi et al. compared WHO G1 and G2 PanNETs on CT and reported G2 tumors tend to be larger (>2.0 cm), with hepatic metastasis and with non-hyperattenuating relative to the pancreatic parenchyma during portal venous phase, with an accuracy to distinguish G2 from G1 tumor with these four features was 82% [11], which is close to the 79% accuracy found in our study, although we also included G3 tumors and PanNEC among the higher-grade tumors. We did not include tumor size in our SVM conventional CT imaging model because this information is already captured by the radiomics features. Adding this feature to the conventional CT imaging model would have likely improved its accuracy.

Enhancement patterns of PanNETs have also been related to tumor grade. D’Assignies et al. reported that tumor blood flow values assessed with dynamic perfusion CT were significantly higher in G1 tumors than G2 or G3 tumors classified by the 2010 WHO classification and correlated with intratumoral microvascular density [14]. Tsukamoto et al. studied contrast washout patterns of PanNETs and found that PanNETs with predominant washout pattern (at least 60 HU washout from peak enhancement in the pancreatic arterial phase to the equilibrium phase) were correlated with significantly lower Ki-67 index compared to PanNETs with persistent enhancement pattern [30].

The results of our study are consistent with those of prior studies regarding conventional CT imaging features for predicting higher-grade tumors. In our study, statistical differences between G1 and higher grades were found for these features with the exception of the HU difference of tumor between arterial and venous phases. The selection of this feature by the logistic regression could be the result of this feature adding valuable information when combined with the other selected features. Interestingly, among enhancement characteristics, except for this feature, all enhancement characteristics among the top 10 features to predict higher-grade PanNENs in our study were obtained from the venous phase.

The use of radiomics to predict tumor grade of PanNETs has emerged with promising results [31,32]. Canellas et al. assessed CT texture analysis of PanNET aggressiveness and reported that texture parameter entropy was predictive of G2/G3 tumors [12]. D’Onofrio et al. assessed CT attenuation of PanNETs and CT texture analysis in 31, 52, and 17 G1, G2, and G3 PanNENs classified by the 2010 WHO classification system and reported that entropy resulted in statistically significant difference between G1 and G3 and between G2 and G3 tumors [33]. They also reported that mean CT attenuation is not different between the tumor grades, but the ratio of CT attenuation of the tumor to adjacent structures (pancreatic parenchyma, aorta, and portal vein) was statistically different between G1 and G3 tumors and G2 and G3 tumors. Gu et al. developed a comprehensive model integrating the tumor margin and CT radiomics signature for predicting G1 and G2/3 tumors classified based on the 2010 WHO system in 104 PanNET cases and reported sensitivity of 86.7%, specificity 89.5%, and accuracy of 88.2% in the validation cohort using CT images obtained from another center [34]. Bian Y et al. assessed 102 patients with non-functioning G1 and G2 PanNETs and reported CT radiomics score using LASSO methods to select radiomics features extracted from portal venous phase CT. In so doing, this achieved a sensitivity of 94.0% and specificity of 63.5% in identifying G2 tumors [18]. Zhao et al. assessed 99 patients with non-functioning PanNETs, and radiomics developed with a combination of nonenhanced and portal venous phase CT achieved a sensitivity of 90.9% and specificity of 88.9% in identifying G2 tumors, though most of the selected features were obtained on the portal venous phase [21]. Higher performance of these models could be explained by a higher number of G3 tumors in the study by Gu et al. [34], the absence of a validation cohort in the study by Bian et al. [18], and considering only non-functional PNET combined with less conventional radiomics features (CoLlAGe and Laws filters) in the study by Zhao et al. [21].

Our study has the advantage of exploring a broader range of radiomics features, including those extracted from filtered images that enhance edges and that were recently introduced in the IBSI guidelines [25]. Out of our top 10 radiomics features, 8 features are derived from the tumor, while 2 are from the full pancreas; 3 were edge features, and 7 were from the original images. In total, 4 of the top 10 radiomics features were original shape features, including 2 original shape features of full pancreas (original shape least axis length and original shape flatness). Only one selected feature is associated with the arterial phase. Even though it is difficult to provide a precise biological interpretation for each individual radiomics feature, the predominance of tumor-derived features suggests that alterations in intra-tumor heterogeneity and morphology are linked to the underlying tumor biology [35,36,37,38]. Combining conventional CT imaging features with CT radiomics features in our SVM model improved the sensitivity to detect higher-grade tumors compared to radiomics features only, which may be due to adding extra-pancreatic conventional CT imaging features such as lymph node metastasis and CT attenuation ratio of the tumor to the pancreas parenchyma, aorta, portal vein that may compensate the technical variability between examinations and between the patients. The combined model with conventional CT imaging, radiomics, and clinical features achieved a high sensitivity (94%) but a moderate specificity (69%). High sensitivity to detect higher-grade tumors would help to reduce the potential risk to misclassify higher-grade tumors as G1 results in conservative management of higher-grade tumors.

There are several limitations to our study. First, it was a retrospective study, and a limited number of cases were analyzed. Especially, a limited number of higher-grade PanNENs were included compared to G1 PanNETs, and we were unable to assess subgroups of G2 tumors with lower and higher Ki-67 index. Also, we combined G2 and G3 PanNETs and PanNEC for analysis. While we did this because some grade 1 PanNETs can be safely observed, and therefore identifying these tumors is clinically important, it means we cannot comment on distinguishing other grades. Second, CT was performed with CT scanners from the same manufacturer and the same protocol, and we were unable to test CT images using other scanners and different protocols. External validation of our models’ performance on an independent dataset is required. Third, there are two cases in our cohort in which tumor grade was determined by FNA, not surgical resection. The proliferation rate in some PanNETs is heterogeneous, and we cannot rule out a higher-grade component in these tumors [3]. Also, we included cases of functioning PanNETs in our cohort. Although functionality may impact prognosis, the biologic behavior of most functioning PanNETs is defined by the grade and stage of the tumor.

## 5. Conclusions

In conclusion, the model based solely on conventional CT imaging features shows higher specificity in identifying patients with PanNENs higher than grade 1 compared to the radiomics model. On the other hand, the radiomics model has higher sensitivity. Combining conventional CT imaging, radiomics, and clinical features yields the best-performing model, indicating that these three categories of features are not redundant and provide complementary information to the model. Such a model may be useful screening tool to detect higher-grade PanNENs.

## Figures and Tables

**Table 1:** List and description of conventional CT imaging features and enhancement pattern.

Features Name	Description	Categorical
Arterial hypo	Hypoattenuating tumor relative to normal pancreas in arterial phase	Yes
Venous hypo	Hypoattenuating tumor relative to normal pancreas in venous phase	Yes
Margin ill defined	Ill-defined tumor margin	Yes
Upstream MPD more than 3 mm	Main pancreatic duct more than 3 mm upstream to tumor	Yes
CBD more than 1 cm mass or stent	Common bile duct more than 1 cm by tumor or presence of CBD stent	Yes
Upstream atrophy	Subjective pancreatic atrophy upstream to tumor	Yes
Exophytic more than 50% volume	≥50% of tumor volume protruding from expected pancreatic contour	Yes
Liver mets	Presence of liver mass(es) suspicious for metastasis	Yes
LN mets	Presence of abnormal abdominal lymph node(s) suspicious for metastasis	Yes
Vascular involvement	Presence of involvement of major peripancreatic vessels	Yes
Calcification	Presence of calcification within the tumor	Yes
Cystic	Well-defined area(s) of homogeneous fluid attenuation within tumor	Yes
Necrotic	Ill-defined nonenhancing area(s) of low attenuation within tumor	Yes
Arterial Tumor HU	Attenuation of solid component of tumor in arterial phase	No
Arterial Pancreas HU	Attenuation of normal pancreas in arterial phase	No
Arterial Aorta HU	Attenuation of abdominal aorta in arterial phase	No
Venous Tumor HU	Attenuation of solid component of tumor in venous phase	No
Venous Pancreas HU	Attenuation of normal pancreas in venous phase	No
Venous Aorta HU	Attenuation of abdominal aorta in venous phase	No
Venous Portal Vein HU	Attenuation of main portal vein in venous phase	No
Arterial Tumor Aorta HU Ratio	Tumor attenuation (HU)/aortic attenuation (HU) in arterial phase	No
Venous Tumor Aorta HU Ratio	Tumor attenuation (HU)/aortic attenuation (HU) in venous phase	No
Arterial Tumor Pancreas HU Ratio	Tumor attenuation (HU)/pancreas attenuation (HU) in arterial phase	No
Venous Tumor Pancreas HU Ratio	Tumor attenuation (HU)/pancreas attenuation (HU) in venous phase	No
Venous Tumor Portal Vein HU Ratio	Tumor attenuation (HU)/portal vein attenuation (HU) in venous phase	No
Arterial Tumor Venous Portal Vein HU Ratio	Tumor attenuation (HU) in arterial phase/portal vein attenuation (HU) in venous phase	No
Arterial Tumor Venous Tumor HU Ratio	Tumor attenuation (HU) in arterial phase/tumor attenuation (HU) in venous phase	No
Arterial Tumor Venous Tumor HU Dif	Tumor attenuation (HU) in arterial phase/tumor attenuation (HU) in venous phase	No

**Table 2:** Patients and tumors’ characteristics.

		Training	Testing	All
Patients		93 *	42 *	133 *
Tumors		96	42	138
Grade				
PanNET				
	Grade 1	62 (64%)	26 (62%)	88 (63%)
	Grade 2	30 (33%)	15 (36%)	45 (33%)
	Grade 3	1 (1%)		1 (1%)
PanNEC		3 (3%)	1 (2%)	4 (3%)
Sex				
	Female	48 (52%)	24 (57%)	70 (54%)
	Male	47 (48%)	18 (43%)	63 (46%)
Surgery				
	No (biopsy only)	2 (2%)	0 (0%)	2 (1%)
	Yes	91 (98%)	42 (100%)	136 (99%)
Location				
	Head	27 (28%)	13 (31%)	40 (29%)
	Body	21 (22%)	4 (10%)	25 (18%)
	Tail	43 (45%)	23 (55%)	66 (48%)
	Neck	1 (1%)	1 (2%)	2 (1%)
	Uncinate	3 (3%)	1 (2%)	4 (3%)
	Diffuse	1 (1%)	0 (0%)	1 (1%)
Patient with incidental cyst				
	No	91 (95%)	40 (95%)	131 (95%)
	Yes	5 (5%)	2 (5%)	7 (5%)
Age				
	Median [range]	60.3 [23.2–82.2]	61.6 [21.5–83.4]	60.6 [21.5–83.4]
Functional Type				
	Nonfunctional	20 (21%)	10 (24%)	30 (22%)
	Serotonin	2 (2%)	2 (5%)	4 (3%)
	Insulinoma	6 (6%)	2 (5%)	8 (6%)
	Unknown	68 (70%)	28 (67%)	95 (69%)
Tumor Focality				
	Unifocal	85 (89%)	41 (98%)	126 (91%)
	Multifocal	11 (11%)	1 (2%)	12 (9%)

* The number of patients in the training and testing sets do not add up to the total number of patients because the split was done at the tumor level.

**Table 3:** Performance of SVM model using CT imaging features, clinical data, and radiomics features on the testing set.

	Accuracy	Sensitivity	Specificity	Precision	F1-Score
**SVM Radiologist**	0.79 (0.67–0.90)	0.75 (0.53–0.94)	0.81 (0.64–0.95)	0.71 (0.50–0.92)	0.73 (0.55–0.87)
**SVM Radiologist + clinical**	0.76 (0.64–0.88)	0.69 (0.45–0.92)	0.81 (0.63–0.94)	0.69 (0.45–0.91)	0.69 (0.47–0.85)
**Radiomics Score**	0.69 (0.54–0.83)	0.94 (0.80 – 1.0)	0.46 (0.34–0.74)	0.56 (0.38–0.74)	0.70 (0.50–0.84)
**SVM Radiomics**	0.69 (0.55–0.81)	0.88 (0.69–1.0)	0.58 (0.38–0.77)	0.56 (0.36–0.76)	0.68 (0.50–0.84)
**SVM Radiomics + Clinical**	0.74 (0.62–0.86)	0.94 (0.79–1.0)	0.62 (0.42–0.79)	0.60 (0.41–0.81)	0.73 (0.57–0.87)
**SVM Radiomics + Radiologist**	0.71 (0.57–0.86)	0.94 (0.80–1.0)	0.58 (0.39–0.76)	0.58 (0.38–0.77)	0.71 (0.53–0.85)
**SVM Radiomics + Radiologist + Clinical**	0.79 (0.64–0.90)	0.94 (0.78–1.0)	0.69 (0.52–0.87)	0.65 (0.45–0.84)	0.77 (0.60–0.91)

The 95% confidence intervals are shown in parentheses. SVM radiologist: Conventional CT imaging features assessed by radiologists. Clinical: Clinical data (age, gender, and tumor location).

## Data Availability

The datasets analyzed in this study are not publicly available due to privacy or ethical restrictions.

## Supplementary Materials

The following supporting information can be downloaded at: https://www.mdpi.com/article/10.3390/cancers17061047/s1, Figure S1: (A) SVM model performance using conventional CT imaging features and clinical data. (B) SVM model performance using CT radiomics features and clinical data. Figure S2: Description of the selected edge features. Figure S3. (A) SVM model performance using conventional CT imaging features and clinical data for small (≤2 cm) tumors. (B) SVM model performance using CT radiomics features and clinical data for small (≤2 cm) tumors. (C) SVM model performance using conventional CT imaging features, CT radiomics features, and clinical data for small (≤2 cm) tumors. SVM radiologist: Conventional CT imaging features assessed by radiologists. Clinical: Clinical data (gender, age, and tumor location).

## Abbreviations

The following abbreviations are used in this manuscript:

CT	Computed tomography
PanNET	Pancreatic neuroendocrine tumor
PanNEN	Pancreatic neuroendocrine neoplasm
PanNEC	Pancreatic neuroendocrine cancer
SVM	Support vector machine
HPF	High power field
WHO	World Health Organization
MPD	Main pancreatic duct
CBD	Common bile duct
HU	Hounsfield units
ROI	Region of interest
GLCM	Gray-level co-occurrence matrix
GLDM	Gray-level differences matrix
GLRLM	Gray-level run-length matrix
GLSZM	Gray-level size-zone matrix
NGTDM	Gray-tone difference matrix
ROC	Receiver operating characteristic
AUC	Area under the curve
