# Supplementary material for: Diagnostic Performance of Combined Conventional CT Imaging Features and Radiomics Signature in Differentiating Grade 1 Tumors from Higher-Grade Pancreatic Neuroendocrine Neoplasms
Source: Cancers (Basel). 2025 Mar 20;17(6):1047. doi: 10.3390/cancers17061047 (PMC11941307; doi:10.3390/cancers17061047)
Supplement: Supplementary file 1 [file cancers-17-01047-s001.zip › cancers-3487023-supplementary.pdf]

(A)

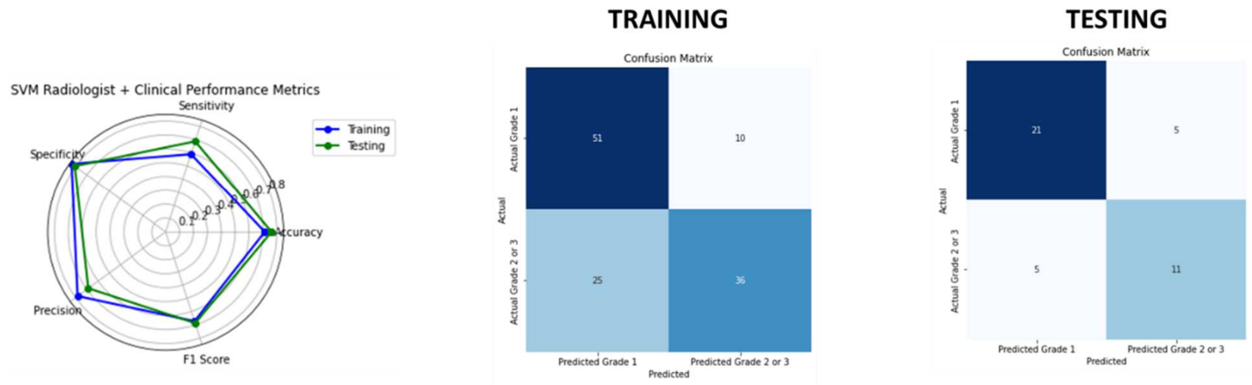

(B)

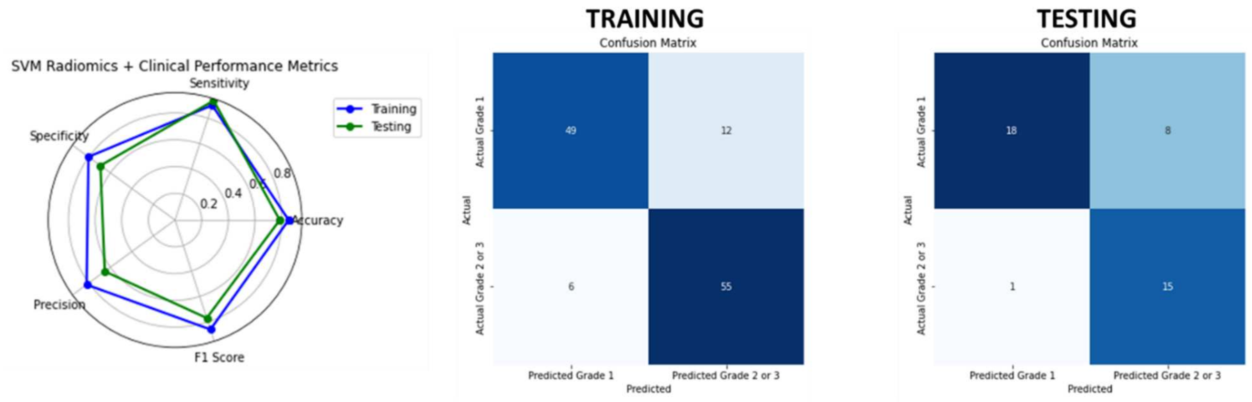

Figure S1. (A) SVM model performance using conventional CT imaging features and clinical data. (B) SVM model performance using CT radiomics features and clinical data. SVM Radiologist: Conventional CT imaging features assessed by radiologists. Clinical: Clinical data (gender, age, tumor location)

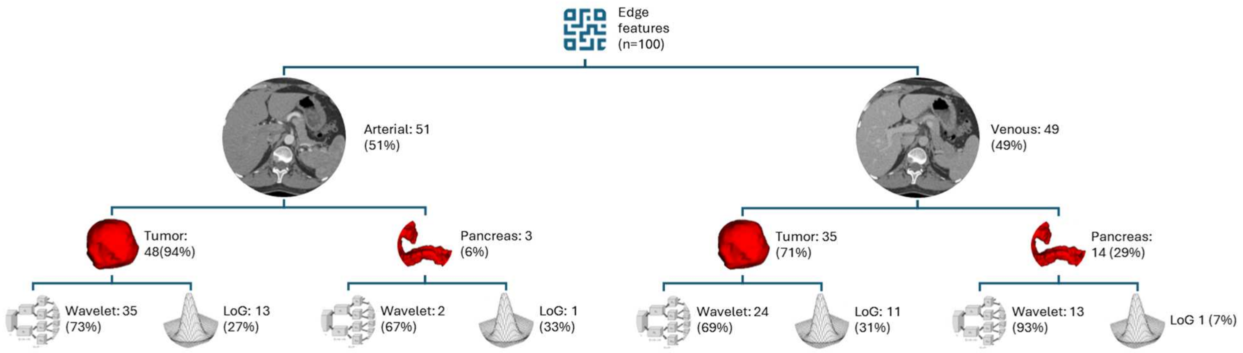

Figure S2. Description of the selected edge features.

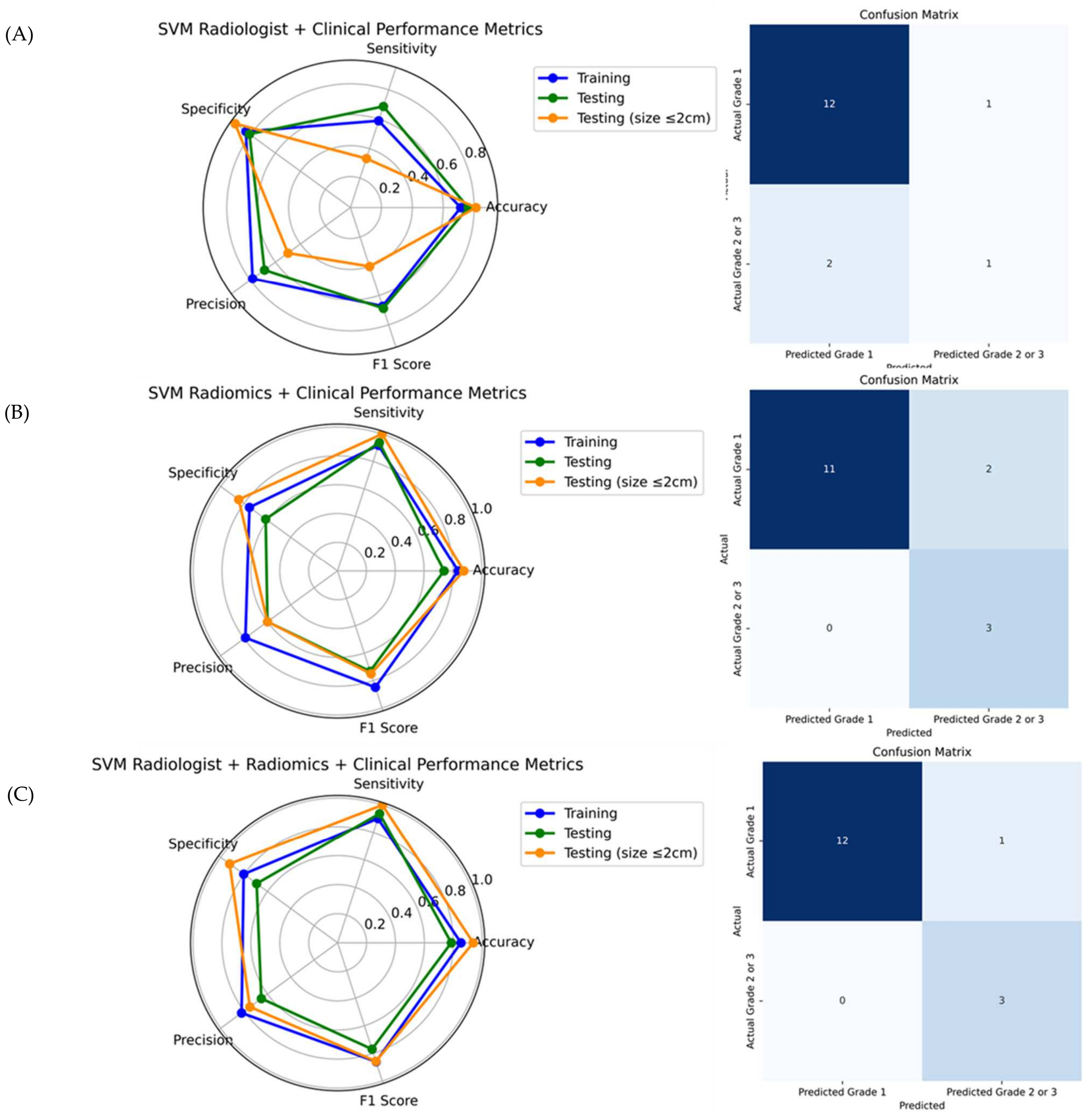

Figure S3. (A) SVM model performance using conventional CT imaging features and clinical data for small (≤ 2 cm) tumors (B) SVM model performance using CT radiomics features and clinical data for small (≤ 2 cm) tumors (C) SVM model performance using conventional CT imaging features, CT radiomics features, and clinical data for small (≤ 2 cm) tumors.

SVM Radiologist: Conventional CT imaging features assessed by radiologists.
Clinical: Clinical data (gender, age, tumor location)
